# Seasonality of Back Pain in Italy: An Infodemiology Study

**DOI:** 10.3390/ijerph18031325

**Published:** 2021-02-01

**Authors:** Jacopo Ciaffi, Riccardo Meliconi, Maria Paola Landini, Luana Mancarella, Veronica Brusi, Cesare Faldini, Francesco Ursini

**Affiliations:** 1 Medicine and Rheumatology Unit, IRCCS Istituto Ortopedico Rizzoli (IOR), 40136 Bologna, Italy; riccardo.meliconi@ior.it (R.M.); luana.mancarella@ior.it (L.M.); veronica.brusi@ior.it (V.B.); francesco.ursini2@unibo.it (F.U.); 2 Department of Biomedical and Neuromotor Science (DIBINEM), University of Bologna, 40125 Bologna, Italy; cesare.faldini@ior.it; 3 Scientific Direction, IRCCS Istituto Ortopedico Rizzoli (IOR), 40136 Bologna, Italy; mariapaola.landini@ior.it; 4 1st Orthopaedic and Traumatologic Clinic, IRCCS Istituto Ortopedico Rizzoli (IOR), 40136 Bologna, Italy

**Keywords:** back pain, Google Trends, infodemiology, seasonality, Wikipedia

## Abstract

Background: E-health tools have been used to assess the temporal variations of different health problems. The aim of our infodemiology study was to investigate the seasonal pattern of search volumes for back pain in Italy. Methods: In Italian, back pain is indicated by the medical word “lombalgia”. Using Google Trends, we selected the three search terms related to “lombalgia” with higher relative search volumes (RSV), (namely, “mal di schiena”, “dolore alla schiena” and “dolore lombare”), representing the semantic preferences of users when performing web queries for back pain in Italy. Wikipedia page view statistics were used to identify the number of visits to the page “lombalgia”. Strength and direction of secular trends were assessed using the Mann–Kendall test. Cosinor analysis was used to evaluate the potential seasonality of back pain-related RSV. Results: We found a significant upward secular trend from 2005 to 2020 for search terms “mal di schiena” (τ = 0.734, *p* < 0.0001), “dolore alla schiena” (τ = 0.713, *p* < 0.0001) and “dolore lombare” (τ = 0.628, *p* < 0.0001). Cosinor analysis on Google Trends RSV showed a significant seasonality for the terms “mal di schiena” (*p*_cos_ < 0.001), “dolore alla schiena” (*p*_cos_ < 0.0001), “dolore lombare” (*p*_cos_ < 0.0001) and “lombalgia” (*p*_cos_ = 0.017). Cosinor analysis performed on views for the page “lombalgia” in Wikipedia confirmed a significant seasonality (*p*_cos_ < 0.0001). Both analyses demonstrated a peak of interest in winter months and decrease in spring/summer. Conclusions: Our infodemiology approach revealed significant seasonal fluctuations in search queries for back pain in Italy, with peaking volumes during the coldest months of the year.

## 1. Introduction

In the dynamic environment of the web, the term *e-health* has been broadly used since late 1990s to encompass all aspects of the Internet and medicine [1]. Over the years, information and communication technology (ICT) for health-related matters has become an important source of implementation for traditional health resources [2]. E-health can facilitate access to services, reduce healthcare costs and improve the quality of patient care [3,4]. Technological advancements offered to policy makers, healthcare professionals and researchers represent an opportunity to stimulate positive health behaviours, promote prevention and simplify retrieval of information about diseases or treatments [5,6,7]. As of July 2020, 59% of the global population, almost 4.57 billion people, were actively using the Internet [8]. As shown in surveys conducted in the United States and Europe, searching the Internet for health information is becoming even easier with the rapidly spreading use of health apps on mobile devices [9]. At the same time, the huge amount of data generated can be collected, stored and communicated over the web to assist decision makers in tailoring precision medicine [10]. The concept of analysing Internet data to predict patterns of disease distribution is known as infodemiology, a term coined in the early 2000s, when the pioneering work of Professor Gunther Eysenbach showed the correlation between Google search volumes for key terms related to influenza and the number of influenza cases occurring one week later [11,12]. This was among the earliest demonstrations of how patterns of search queries and their changes over time reflected public interest towards a specific health-related topic. In the following years, data from Google searches obtained through the Google Trends interface have been used to investigate seasonal variations of different health problems such as restless leg syndrome [13,14], multiple sclerosis [15], depression [16] and bruxism [17]. More specifically, in the field of musculoskeletal diseases, Google Trends has been used to evaluate global interest and seasonality of searches for rheumatoid arthritis [18,19], systemic lupus erythematosus [20,21], ankylosing spondylitis [22], osteoarthritis [23], gout [24] and fibromyalgia [25]. Moreover, seasonality of common joint symptoms such as foot and ankle pain [26] or knee pain [27] has been assessed. Interestingly, recent studies outlined how, amongst searches for painful conditions, back pain was one of the top queries, raising the hypothesis of seasonal variations in search trends [28,29]. Back pain is indeed an extremely common complaint [30], to the point that in 2015 the estimated prevalence of activity-limiting low back pain was 7.3% globally, corresponding to 540 million people suffering from the condition [31]. These numbers imply that back pain, and in particular low back pain, now represents the first cause of disability in the world [31]. Back pain is experienced by individuals of all ages and has a considerable impact on global public health [32]. Therefore, as expected, the volume of web searches for back pain have increased worldwide in the last years [28] and, since technological advancements have allowed people to seek on the Internet healthcare information and advice about medicine and treatments, using Internet data to understand the interest in back pain could be of relevance for healthcare professionals. As of October 2020, Google led the search engine market in Italy with 96.3% of share [33]. Therefore, the analysis of search volumes performed with Google has a solid basis supporting its reliability. However, besides Google Trends, data also accumulated during Internet search activities on Wikipedia pages have been used for research purposes and to build epidemiological models [34,35]. An increasing number of investigations have used Google Trends and Wikipedia page view data to assess public interest in different health conditions and their seasonal variations, providing novel and complementary methods to integrate information from traditional sources [36,37,38,39]. Therefore, we decided to conduct an ecological infodemiology study using Internet data to track search patterns and identify variability in public interest for back pain in Italy, specifically aiming at determining periods with highest and lowest search popularity. Potential clinical implications of our study are primarily related to the application of retrieved data to guide preventative interventions for low back pain and to develop specific health programmes to effectively manage and reduce the burden of the condition.

## 2. Materials and Methods

### 2.1. Google Trends Data Availability

Google Trends (Google Inc., Menlo Park, CA, USA) [40] is a free instrument which allows researchers to explore data on the frequency of query strings utilized by users when performing a search in Google. Data are available from January 2004. To improve comparability between search terms, Google Trends data are adjusted to the overall volume of searches for a given time frame and geographical location and provided as a relative search volume (RSV) ranging from 0 to 100 [41]. To reduce selection bias, Google Trends automatically excludes duplicate queries performed by the same user (i.e., identical IP address) within a short period of time [41]. In Google Trends, words or phrases can be searched either as search terms (ST, search queries matching for all terms, similar to the application of the “AND” Boolean operator) or “topics” (TP), a group of related terms that share the same concept in any language [41]. Furthermore, each query in Google Trends generates a table of related searches, ranked for either percentage increase in RSV or relative frequency (0–100) [41]. The data obtained can be exported as comma-separated values (.CSV) files for further manipulation and analysis.

### 2.2. Wikipedia Page View Data Availability

Wikipedia page view statistics [42] is a free instrument available for all Wikipedia pages, which allows researchers to ascertain how many people have visited an article during a given time frame [43]. Data on visit counts are available from July 2015 and can be generated on a daily or monthly basis and according to language-specific Wikipedia projects or user platforms (desktop, mobile application, mobile web) [43]. Different from Google Trends, each time a user visits or reloads a Wikipedia page, a new count is generated [43]. The data retrieved can be exported as comma-separated values (.CSV) files for further manipulation and analysis.

### 2.3. Search Process and Data Retrieval

On 26 April 2020, we performed a Google Trends search to identify queries in the “health” category related to back pain in Italy on a month-by-month basis, from January 2004 to April 2020. To select the search terms or topic that best fitted the user preference in performing back pain-related searches, we followed a systematic approach. First of all, we searched the ST “lombalgia” (medical term used in Italy to describe low back pain) and “dolore” (corresponding to generic “pain” in the Italian language) and explored related searches after ranking for their RSV. Subsequently, we selected the three search terms that resulted in the higher RSVs (“mal di schiena”, “dolore alla schiena”, “dolore lombare”) while at the same time reflecting a precise intention of the user to retrieve information on back pain. Furthermore, we performed a Wikipedia page view search to retrieve the number of visits to the page “Lombalgia” [44] from 1 July 2015 to 30 April 2020. Data were retrieved for searches from all platforms on the https://it.wikipedia.org/wiki/Pagina_principale.

### 2.4. Statistical Analysis

Reporting of results was compliant with a previously published Checklist for the Documentation of Google Trends Use [38]. To graphically evaluate the temporal trend for individual search terms, we plotted time series data using the Locally Weighted Scatter Plot Smoothing (LOESS) function [45]. LOESS is a nonparametric method where least squares regression is performed in local neighbourhood subsets of data, smoothing numerical vectors. LOESS curves are mainly used to reveal trends and cycles in data that might be difficult to model with a parametric curve. To assess the significance of temporal trends in back pain-related Google searches, the Mann–Kendall test [46,47] was used. The Mann–Kendall is a nonparametric test to statistically assess if there is a monotonic upward or downward trend of the variable of interest over time, and it is based on the difference in signs between earlier and later data points of a time series. The strength and direction of the trend is represented by the τ (tau) value (positive = upward, negative = downward). Cosinor analysis was used to evaluate the potential seasonality of back pain-related RSV. Cosinor-based techniques have been extensively adopted to analyse time series in chronobiology [48]. In short, cosinor analysis fits a sinusoid to the experimental data according to the following equation:

Y(t) = M + Acos(2πt/τ + ϕ) + e(t)(1)

where M is the MESOR (Midline Statistic of Rhythm, a rhythm-adjusted mean), A is the amplitude (a measure of the amplitude of the variation of the peak from the baseline), ϕ is the acrophase (a measure of the time of the peak), τ is the period (duration of one cycle) and e(t) is the error term. Since the included variables were continuous, a Poisson model was fitted. The cosine model consists of sine and cosine terms that together identify the sinusoid. Therefore, it contains two *p*-values, one of which was shown as recommended. To control the false discovery rate caused by multiple testing, the criterion of significance was set at *p* < 0.025. Data processing and analyses were conducted using the R software, V.3.4.4 (R Core Team, Auckland, New Zealand) using the “season”, “kendall” and “ggplot2” packages.

### 2.5. Ethical Considerations

Ethical approval was deemed unnecessary because data were provided in aggregate form with no person-identifiable information; further explanations on personal data protection are available on the Google privacy policy website [49].

## 3. Results

### 3.1. Secular Trend of Google Searches for Back Pain

**Figure 1:**
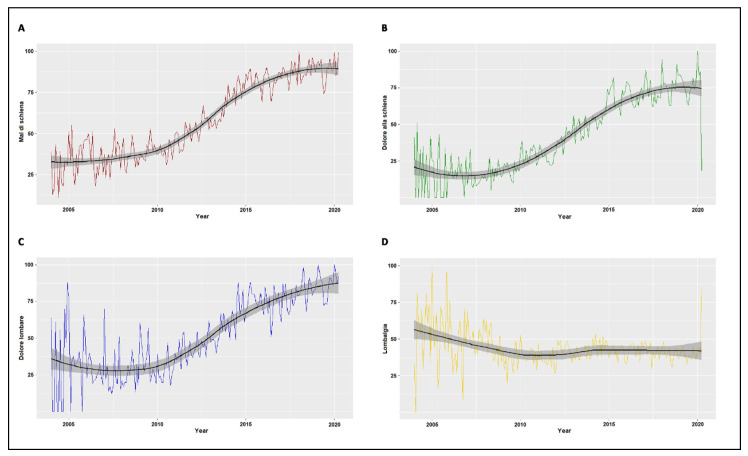
Time series plot Locally Weighted Scatter Plot Smoothing (LOESS) of relative search volumes (RSV) for the search terms “mal di schiena” (**A**), “dolore alla schiena” (**B**), “dolore lombare” (**C**), “lombalgia” (**D**) from January 2004 to April 2020.

Visual inspection of the time series plot with LOESS smoothing suggested an increasing secular trend of RSV for the search terms “mal di schiena” (Figure 1A), “dolore alla schiena” (Figure 1B) and “dolore lombare” (Figure 1C). No clearly identifiable trend was evident for the search term “lombalgia” (Figure 1D). Mann–Kendall analysis confirmed a significant upward secular trend for the search terms “mal di schiena” (τ = 0.734, *p* < 0.0001), “dolore alla schiena” (τ = 0.713, *p* < 0.0001) and “dolore lombare” (τ = 0.628, *p* < 0.0001) and a significant, although less pronounced downward trend for the search term “lombalgia” (τ = −0.128, *p* = 0.009). 

### 3.2. Analysis of Seasonality

**Figure 2:**
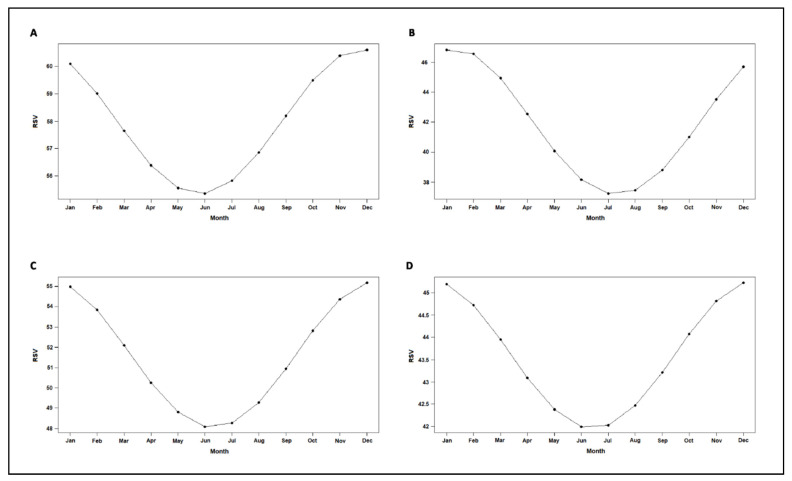
Seasonal fluctuations for the terms “mal di schiena” (**A**), “dolore alla schiena” (**B**), “dolore lombare” (**C**) and “lombalgia” (**D**) based on Cosinor analysis on Google Trends relative search volumes (RSV) from 196 monthly observations from January 2004 to April 2020.

The cosinor analysis on RSV obtained from Google Trends searches based on 196 monthly observations showed a significant seasonality for the search terms “mal di schiena” (A = 3.63, ϕ = 11.8, low point month = 5.8, *p*_cos_ < 0.001) (Figure 2A), “dolore alla schiena” (A = 5.12, ϕ = 1.3, low point = 7.3, *p*_cos_ < 0.0001) (Figure 2B), “dolore lombare” (A = 3.73, ϕ = 12.3, low point = 6.3, *p*_cos_ < 0.0001) (Figure 2C) and “lombalgia” (A = 1.69, ϕ = 12.4, low point = 6.4, *p*_cos_ = 0.017) (Figure 2D), demonstrating a peak in RSV for back pain-related search terms in the central winter months and a decrease at the beginning of summer.

Furthermore, we analysed the monthly views for the page “lombalgia” in Wikipedia. Cosinor analysis, performed on the available time frame (July 2015–April 2020, 58 observations) confirmed a significant seasonality (A = 117.33, ϕ = 10.2, low point = 4.2, *p*_cos_ < 0.0001) with a peak in early winter and a decrease in spring.

## 4. Discussion

E-health tools are increasingly being used in the healthcare sector by both patients and providers, with broad potential applications [50]. Low back pain is a multifactorial condition caused by the interaction of genetic, physical, psychological and occupational factors, with a complex pathophysiology [51]. The aim of our study was to investigate the seasonal variation in back pain-related searches in Italy through an infodemiology approach. Results obtained from the analysis of Google Trends search volumes and Wikipedia page view statistics demonstrated a significantly peaking interest towards back pain during the winter months.

Several mechanisms may account for the observed seasonality of back pain. First of all, it is already known and commonly accepted that meteorological variables such as temperature and humidity can modulate chronic pain [52,53,54,55,56,57]; in particular, in individuals with chronic low back pain, specific weather conditions (e.g., ambient temperature and vapor pressure) are assumed to affect pain perception [58]. Although these effects are small in magnitude, considering meteorological parameters as potential modulators of pain perception entails possible implications for clinical management of patients with chronic low back pain. To this purpose, environmental variables can be manipulated in the home or work setting in order to achieve optimal conditions for minimizing the impact on musculoskeletal pain.

On the other side, literature findings regarding the association between weather and back pain are still inconsistent, with studies showing no effects of meteorological parameters on the onset of back pain or on the intensity of symptoms during an exacerbation [59,60]. At polar latitudes, less chronic musculoskeletal pain has been described in winter compared to summer [61], but the variation, although statistically significant, was minimal, with questionable clinical relevancy. Moreover, hospital admissions for back pain exacerbations were found to be unrelated to average temperature or rainfall [62].

Physical activity is a well-recognized barrier to the development of musculoskeletal pain. A recent meta-analysis of cross-sectional studies demonstrated that people engaged in medium level physical activity have a 10% lower risk of low back pain when compared to those engaged in low level physical activity [63]. A clear seasonality has been demonstrated in average levels of physical activity, with leisure-time energy expenditure approximately 15–20% higher during spring and summer [64]. On this basis, another potential explanation of the observed seasonal variation in back pain may be attributable to a more sedentary lifestyle during the winter months. Complementarily, cold season is associated with a change in eating patterns (resulting in a higher caloric intake), contributing to an increase in body weight peaking in winter [65]. Obesity, in turn, is a well-recognized risk factor for the development of back pain [66].

Another possible explanation for our findings is the correlation between back pain and vitamin D status. It is well established that vitamin D plasma levels are influenced by seasonal variations, with highest values reported in summer [67]. A relationship between vitamin D status and low back pain has been extensively demonstrated in literature, with stronger associations observed in younger women and those with markedly reduced levels of vitamin D [68]. Severity of vitamin D deficiency has also been postulated to be causally associated with chronic low back pain, with increasing intensity of symptoms in patients with lower vitamin D levels [69,70].

Furthermore, it is possible to speculate that seasonal fluctuations observed in the RSV for back pain in Italy may be influenced also by other factors, for instance the differences in footwear used by the population between winter and summer, with potential implications on the development of postural back pain symptoms [71,72].

However, it could be argued that the retrieved seasonal pattern might be explained by the highest use of the web during winter months when people spend more time at home. Anyway, results obtained through the Google Trends platform represent relative volumes normalized on overall search engine traffic and therefore less likely to be influenced by reduced web usage; furthermore, it should be pointed out that several earlier studies focusing on different health topics showed seasonal variations with Internet searches peaking in summer [13,24,26,73,74].

Additionally, a limitation of our research, intrinsically related to the nature of infodemiology studies, is that the analysis of Google Trends is only able to capture the search behaviour in a given period of time and geographic area, representing the interest people have in a topic, which in the case of back pain does not necessarily reflect the actual number of individuals experiencing the symptom. As shown by previous evidence, web queries may be influenced by other variables not included in our study. News regarding celebrities may have a significant impact on the public opinion, with a consequent increase in web search volumes [19,75,76]. Moreover, global initiatives to raise awareness on medical conditions may influence Internet search query activity [77] and, in the specific case of low back pain, we can’t exclude that World Spine Day taking place every year on 16 October might have contributed to the increase in interest observed in early winter. The major strengths of our study are the large amount of data, the long observation period and the analysis of two complementary sources, namely, Google Trends and Wikipedia. Nevertheless, some limitations need to be acknowledged. We analysed the seasonality of searches for back pain only in Italy; therefore, our results cannot be generalized to other countries. However, the value of our methodology consisted in the use of common language “everyday words”, requiring a deep knowledge of local semantics for back pain symptoms in different geographical and linguistic areas. Moreover, web data do not allow researchers to gather demographic information about the users, thus precluding the opportunity to stratify our findings in specific subpopulations and analyse differences between men and women or in different age groups. Hence, our results can only be interpreted at a general Italian population level.

## 5. Conclusions

In conclusion, our infodemiology approach revealed significant seasonal fluctuations in search queries for back pain in Italy, with peaking volumes during the coldest months of the year. According to previous literature, weather conditions, physical activity or vitamin D status might explain, at least in part, the observed seasonal pattern. Despite introducing an intriguing hypothesis, our findings should be considered preliminary and require clinical validation; furthermore, possible mechanisms behind the seasonality of back pain need to be further elucidated. If confirmed in future studies, the winter-peaking seasonality of back pain could be exploited for implementing prophylactic strategies, e.g., encouraging seasonally tailored physical activity programs and calorie restriction or promoting recognition and management of vitamin D deficiency when appropriate.

## Data Availability

No new data were created in this study. Data sharing is not applicable to this article because the data presented are openly available on the web.

## References

[1] Eysenbach G. (2001). What is e-health?. J. Med. Internet Res..

[2] Pagliari C., Sloan D., Gregor P., Sullivan F., Detmer D., Kahan J.P., Oortwijn W., MacGillivray S. (2005). What is eHealth (4): A scoping exercise to map the field. J. Med. Internet Res..

[3] Jung M.L., Loria K. (2010). Acceptance of Swedish e-health services. J. Multidiscip. Healthc..

[4] González M.E., Quesada G., Urrutia I., Gavidia J.V. (2006). Conceptual design of an e-health strategy for the Spanish health care system. Int. J. Health Care Qual. Assur..

[5] Srivastava S., Pant M., Abraham A., Agrawal N. (2015). The Technological Growth in eHealth Services. Comput. Math. Methods Med..

[6] Kampmeijer R., Pavlova M., Tambor M., Golinowska S., Groot W. (2016). The use of e-health and m-health tools in health promotion and primary prevention among older adults: A systematic literature review. BMC Health Serv. Res..

[7] Strzelecki A. (2020). Google Medical Update: Why Is the Search Engine Decreasing Visibility of Health and Medical Information Websites?. Int. J. Environ. Res. Public Health.

[8] Global Digital Population as of October 2020. https://www.statista.com/statistics/617136/digital-population-worldwide/.

[9] Bach R.L., Wenz A. (2020). Studying health-related internet and mobile device use using web logs and smartphone records. PLoS ONE.

[10] Pastorino R., De Vito C., Migliara G., Glocker K., Binenbaum I., Ricciardi W., Boccia S. (2019). Benefits and challenges of Big Data in healthcare: An overview of the European initiatives. Eur. J. Public Health.

[11] Eysenbach G. (2006). Infodemiology: Tracking flu-related searches on the web for syndromic surveillance. AMIA Annu. Symp. Proc..

[12] Eysenbach G. (2009). Infodemiology and infoveillance: Framework for an emerging set of public health informatics methods to analyze search, communication and publication behavior on the Internet. J. Med. Internet Res..

[13] O’Keeffe S.T. (2017). Summertime blues? A re-examination of the seasonality of web searches for restless legs and leg cramps. Sleep Med..

[14] Ingram D.G., Plante D.T. (2013). Seasonal trends in restless legs symptomatology: Evidence from Internet search query data. Sleep Med..

[15] Moccia M., Palladino R., Falco A., Saccà F., Lanzillo R., Brescia Morra V. (2016). Google Trends: New evidence for seasonality of multiple sclerosis. J. Neurol. Neurosurg Psychiatry.

[16] Yang A.C., Huang N.E., Peng C.K., Tsai S.J. (2010). Do seasons have an influence on the incidence of depression? The use of an internet search engine query data as a proxy of human affect. PLoS ONE.

[17] Kardeş S., Kardeş E. (2019). Seasonality of bruxism: Evidence from Google Trends. Sleep Breath.

[18] Wu G.C., Tao S.S., Zhao C.N., Mao Y.M., Wu Q., Dan Y.L., Pan H.F. (2019). Leveraging Google Trends to investigate the global public interest in rheumatoid arthritis. Rheumatol. Int..

[19] Mahroum N., Bragazzi N.L., Sharif K., Gianfredi V., Nucci D., Rosselli R., Brigo F., Adawi M., Amital H., Watad A. (2018). Leveraging Google Trends, Twitter, and Wikipedia to Investigate the Impact of a Celebrity’s Death From Rheumatoid Arthritis. J. Clin. Rheumatol..

[20] Wu G.C., Cao F., Shen H.H., Hu L.Q., Hu Y., Sam N.B. (2019). Global public interest in systemic lupus erythematosus: An investigation based on internet search data. Lupus.

[21] Pan H.F., Wang P., Wu G.C., Zou Y.F., Xu Z., Ye D.Q., Hu W. (2019). Seasonal variation in systemic lupus erythematosus and rheumatoid arthritis: An ecological study based on internet searches. Autoimmun. Rev..

[22] Mei Y.J., Mao Y.M., Cao F., Wang T., Li Z.J. (2020). Using internet search data to explore the global public concerns in ankylosing spondylitis. Postgrad. Med. J..

[23] Jellison S.S., Bibens M., Checketts J., Vassar M. (2018). Using Google Trends to assess global public interest in osteoarthritis. Rheumatol. Int..

[24] Kardeş S. (2019). Seasonal variation in the internet searches for gout: An ecological study. Clin. Rheumatol..

[25] Bragazzi N.L., Amital H., Adawi M., Brigo F., Watad S., Aljadeff G., Amital D., Watad A. (2017). What do people search online concerning the “elusive” fibromyalgia? Insights from a qualitative and quantitative analysis of Google Trends. Clin. Rheumatol..

[26] Telfer S., Woodburn J. (2015). Let me Google that for you: A time series analysis of seasonality in internet search trends for terms related to foot and ankle pain. J. Foot Ankle Res..

[27] Dewan V., Sur H. (2018). Using google trends to assess for seasonal variation in knee injuries. J. Arthrosc. Jt. Surg..

[28] Kamiński M., Łoniewski I., Marlicz W. (2020). “Dr. Google, I am in Pain”-Global Internet Searches Associated with Pain: A Retrospective Analysis of Google Trends Data. Int. J. Environ. Res. Public Health.

[29] Yamaguchi Y., Lee D., Nagai T., Funamoto T., Tajima T., Chosa E. (2020). Googling Musculoskeletal-Related Pain and Ranking of Medical Associations’ Patient Information Pages: Google Ads Keyword Planner Analysis. J. Med. Internet Res..

[30] Hoy D., Bain C., Williams G., March L., Brooks P., Blyth F., Woolf A., Vos T., Buchbinder R. (2012). A systematic review of the global prevalence of low back pain. Arthritis Rheum.

[31] (2016). Global, regional, and national incidence, prevalence, and years lived with disability for 310 diseases and injuries, 1990-2015: A systematic analysis for the Global Burden of Disease Study 2015. Lancet.

[32] Hartvigsen J., Hancock M.J., Kongsted A., Louw Q., Ferreira M.L., Genevay S., Hoy D., Karppinen J., Pransky G., Sieper J. (2018). What low back pain is and why we need to pay attention. Lancet.

[33] Market Share Held by the Leading Search Engines in Italy between September 2019 and September 2020. https://www.statista.com/statistics/623043/search-engines-ranked-by-market-share-in-italy/.

[34] Hickmann K.S., Fairchild G., Priedhorsky R., Generous N., Hyman J.M., Deshpande A., Del Valle S.Y. (2015). Forecasting the 2013–2014 influenza season using Wikipedia. PLoS Comput. Biol..

[35] Sciascia S., Radin M. (2017). What can Google and Wikipedia can tell us about a disease? Big Data trends analysis in Systemic Lupus Erythematosus. Int. J. Med. Inform..

[36] Cervellin G., Comelli I., Lippi G. (2017). Is Google Trends a reliable tool for digital epidemiology? Insights from different clinical settings. J. Epidemiol. Glob. Health.

[37] Brownstein J.S., Freifeld C.C., Madoff L.C. (2009). Digital disease detection—Harnessing the Web for public health surveillance. N. Engl. J. Med..

[38] Nuti S.V., Wayda B., Ranasinghe I., Wang S., Dreyer R.P., Chen S.I., Murugiah K. (2014). The use of google trends in health care research: A systematic review. PLoS ONE.

[39] Mavragani A., Ochoa G., Tsagarakis K.P. (2018). Assessing the Methods, Tools, and Statistical Approaches in Google Trends Research: Systematic Review. J. Med. Internet Res..

[40] Google Trends. https://trends.google.com/.

[41] Google Trends Help Center. https://support.google.com/trends/.

[42] Wikipedia: Page View Statistics. https://tools.wmflabs.org/pageviews/.

[43] Pageviews Analysis—Documentation. https://meta.wikimedia.org/wiki/Pageviews_Analysis.

[44] Wikipedia: Lombalgia. https://it.wikipedia.org/wiki/Lombalgia.

[45] Cleveland W.S., Devlin S.J. (1988). Locally Weighted Regression: An Approach to Regression Analysis by Local Fitting. J. Am. Stat. Assoc..

[46] Mann H.B. (1945). Nonparametric tests against trend. Econometrica.

[47] Kendall M.G. (1975). Rank Correlation Methods.

[48] Cornelissen G. (2014). Cosinor-based rhythmometry. Theor. Biol. Med Model..

[49] Google Privacy & Terms. https://policies.google.com/.

[50] Penedo F.J., Oswald L.B., Kronenfeld J.P., Garcia S.F., Cella D., Yanez B. (2020). The increasing value of eHealth in the delivery of patient-centred cancer care. Lancet Oncol..

[51] Vlaeyen J.W.S., Maher C.G., Wiech K., Van Zundert J., Meloto C.B., Diatchenko L., Battié M.C., Goossens M., Koes B., Linton S.J. (2018). Low back pain. Nat. Rev. Dis. Primers.

[52] Shutty M.S., Cundiff G., DeGood D.E. (1992). Pain complaint and the weather: Weather sensitivity and symptom complaints in chronic pain patients. Pain.

[53] Jamison R.N., Anderson K.O., Slater M.A. (1995). Weather changes and pain: Perceived influence of local climate on pain complaint in chronic pain patients. Pain.

[54] Roth-Isigkeit A., Thyen U., Stöven H., Schwarzenberger J., Schmucker P. (2005). Pain among children and adolescents: Restrictions in daily living and triggering factors. Pediatrics.

[55] Timmermans E.J., van der Pas S., Schaap L.A., Sánchez-Martínez M., Zambon S., Peter R., Pedersen N.L., Dennison E.M., Denkinger M., Castell M.V. (2014). Self-perceived weather sensitivity and joint pain in older people with osteoarthritis in six European countries: Results from the European Project on OSteoArthritis (EPOSA). BMC Musculoskelet. Disord..

[56] Moldofsky H. (1994). Chronobiological influences on fibromyalgia syndrome: Theoretical and therapeutic implications. Baillieres Clin. Rheumatol..

[57] Hawley D.J., Wolfe F., Lue F.A., Moldofsky H. (2001). Seasonal symptom severity in patients with rheumatic diseases: A study of 1,424 patients. J. Rheumatol..

[58] McGorry R.W., Hsiang S.M., Snook S.H., Clancy E.A., Young S.L. (1998). Meteorological conditions and self-report of low back pain. Spine.

[59] Steffens D., Maher C.G., Li Q., Ferreira M.L., Pereira L.S., Koes B.W., Latimer J. (2014). Effect of weather on back pain: Results from a case-crossover study. Arthritis Care Res..

[60] Beilken K., Hancock M.J., Maher C.G., Li Q., Steffens D. (2017). Acute Low Back Pain? Do Not Blame the Weather—A Case-Crossover Study. Pain Med..

[61] Abeler K., Sand T., Friborg O., Bergvik S. (2020). Seasonality in pain, sleep and mental distress in patients with chronic musculoskeletal pain at latitude 69° N. Chronobiol. Int..

[62] Okwerekwu G., Brooks F., Spolton-Dean C., Khurana A., Manoj-Thomas A., Cordell-Smith J. (2015). Is there a seasonal variation of acute admissions for back pain. Spine J..

[63] Alzahrani H., Mackey M., Stamatakis E., Zadro J.R., Shirley D. (2019). The association between physical activity and low back pain: A systematic review and meta-analysis of observational studies. Sci. Rep..

[64] Pivarnik J.M., Reeves M.J., Rafferty A.P. (2003). Seasonal variation in adult leisure-time physical activity. Med. Sci. Sports Exerc..

[65] Ma Y., Olendzki B.C., Li W., Hafner A.R., Chiriboga D., Hebert J.R., Campbell M., Sarnie M., Ockene I.S. (2006). Seasonal variation in food intake, physical activity, and body weight in a predominantly overweight population. Eur. J. Clin. Nutr..

[66] Zhang T.T., Liu Z., Liu Y.L., Zhao J.J., Liu D.W., Tian Q.B. (2018). Obesity as a Risk Factor for Low Back Pain: A Meta-Analysis. Clin. Spine Surg..

[67] Holick M.F. (1995). Environmental factors that influence the cutaneous production of vitamin D. Am. J. Clin. Nutr..

[68] Zadro J., Shirley D., Ferreira M., Carvalho-Silva A.P., Lamb S.E., Cooper C., Ferreira P.H. (2017). Mapping the Association between Vitamin D and Low Back Pain: A Systematic Review and Meta-Analysis of Observational Studies. Pain Physician.

[69] Gokcek E., Kaydu A. (2018). Assessment of Relationship between Vitamin D Deficiency and Pain Severity in Patients with Low Back Pain: A Retrospective, Observational Study. Anesth Essays Res..

[70] Panwar A., Valupadas C., Veeramalla M., Vishwas H.N. (2018). Prevalence of vitamin D deficiency in chronic and subacute low back pain patients in India: A triple-arm controlled study. Clin. Rheumatol..

[71] Vieira E.R., Brunt D. (2016). Does wearing unstable shoes reduce low back pain and disability in nurses? A randomized controlled pilot study. Clin. Rehabil..

[72] Bai D.Y., Yuan Z.G., Shao J.J., Zhu T., Zhang H.J. (2019). Unstable shoes for the treatment of lower back pain: A meta-analysis of randomized controlled trials. Clin. Rehabil..

[73] Liu F., Allan G.M., Korownyk C., Kolber M., Flook N., Sternberg H., Garrison S. (2016). Seasonality of Ankle Swelling: Population Symptom Reporting Using Google Trends. Ann. Fam. Med..

[74] Zhang X., Dang S., Ji F., Shi J., Li Y., Li M., Jia X., Wan Y., Bao X., Wang W. (2018). Seasonality of cellulitis: Evidence from Google Trends. Infect. Drug Resist..

[75] Tijerina J.D., Morrison S.D., Nolan I.T., Parham M.J., Richardson M.T., Nazerali R. (2019). Celebrity Influence Affecting Public Interest in Plastic Surgery Procedures: Google Trends Analysis. Aesthetic Plast. Surg..

[76] Kaleem T., Malouff T.D., Stross W.C., Waddle M.R., Miller D.H., Seymour A.L., Zaorsky N.G., Miller R.C., Trifiletti D.M., Vallow L. (2019). Google Search Trends in Oncology and the Impact of Celebrity Cancer Awareness. Cureus.

[77] Havelka E.M., Mallen C.D., Shepherd T.A. (2020). Using Google Trends to assess the impact of global public health days on online health information seeking behaviour in Central and South America. J. Glob. Health.

